# Telephone-Delivered Interventions for Suicide Prevention in Schizophrenia and Related Disorders: A Systematic Review

**DOI:** 10.3390/healthcare11030432

**Published:** 2023-02-02

**Authors:** Laura Comendador, Ana Isabel Cebrià, Antoni Sanz, Víctor Pérez, Diego Palao

**Affiliations:** 1 Department of Psychiatry and Forensic Medicine, Faculty of Medicine, Universitat Autònoma de Barcelona, 08193 Cerdanyola del Vallès, Spain; 2 Unitat Mixta de Neurociència Traslacional I3PT-INc-UAB, Institut d’Investigació i Innovació Parc Taulí I3PT, Department of Mental Health, University Hospital Parc Taulí, 08208 Sabadell, Spain; 3 Centro de Investigación Biomédica en Red de Salud Mental (CIBERSAM), Instituto de Salud Carlos III, 28029 Madrid, Spain; 4 Department of Clinical and Health Psychology, Faculty of Psychology, Universitat Autònoma de Barcelona, 08193 Cerdanyola del Vallès, Spain; 5 Department of Basic, Developmental and Educational Psychology, Faculty of Psychology, Universitat Autònoma de Barcelona, 08193 Cerdanyola del Vallès, Spain; 6 Stress and Health Research Group (GIES), Universitat Autònoma de Barcelona, 08193 Cerdanyola del Vallès, Spain; 7 Institut de Neuropsiquiatria i Addiccions (INAD), Institut Hospital del Mar d’Investigacions Mèdiques (IMIM), Universitat Autònoma de Barcelona, Parc de Salut Mar, 08003 Barcelona, Spain

**Keywords:** schizophrenia, suicide, telehealth, monitoring, non-pharmacological interventions, psychology, emotions, cognitive symptoms

## Abstract

Background: Suicide is a health problem among patients diagnosed with schizophrenia. Telehealth technology has become an emerging intervention that may offer opportunities to reach this at-risk group. However, to consider the implementation of telehealth systems in the prevention of suicidal behaviors in patients diagnosed with schizophrenia, a review of the evidence is required. The present aim was to explore the effectiveness of telephone-based suicide prevention programs among patients with schizophrenia and related disorders. Methods: A bibliographic search was carried out in the PubMed, PsycInfo, Scopus and Web of Science electronic databases following PRISMA guidelines. Two reviewers performed the selection, data extraction and methodological quality assessment. A total of 352 articles were retrieved, of which five studies met the eligibility criteria. Results: Globally, an adherence was observed ranging from 78 to 100%. Three studies reported a reduction in suicidal ideation and two studies showed a reduction in the risk of relapse observed in the intervention group compared to a control group. Conclusions: In accordance with the limited data available, the use of a telephone contact approach appears to be feasible and effective in schizophrenia patients with suicidal behaviors. The preliminary evidence also suggests that this system appears to reduce suicidal ideation. Further research is required to design evidence-based future interventions and to determine whether this approach can improve patient outcomes.

## 1. Introduction

Schizophrenia is a long-term mental illness associated with considerable mortality and morbidity [1]. Suicide represents one of the leading causes of premature death in patients affected by schizophrenia and a critical global issue that has a major impact on public health [2,3]. For decades, suicide has remained one of the leading causes of preventable death worldwide [4]. Moreover, patients diagnosed with schizophrenic disorder are a suicide risk group, especially during chronic relapses and the first months following hospital discharge [5,6,7]. Hence, it appears to be imperative to adopt specific measures to reduce suicidal behaviors in patients with schizophrenia and related disorders.

Telemedicine helps increase access to services for at-risk populations and provides opportunities to augment mental health services [8]. Telehealth has become more interactive, affordable and widely available to healthcare providers in addressing chronic medical conditions [8]. These telecommunication-based interventions—which include videoconferencing, telephone contacts and internet-based programs [9,10]—provide a reliable approach to monitoring the risk of suicide in patients with schizophrenia spectrum disorders [3].

A growing body of research is emerging on the effectiveness of telehealth interventions in patients admitted to the emergency department (ED) for suicidal behavior. Fleischmann et al. [11] reported significantly fewer suicide attempts and differences in the number of suicide deaths in patients who received telephone contacts for 18 months in comparison to a control group. Vaiva et al. [12] reported significantly fewer suicide attempts in participants receiving telephone contact one month after a suicide attempt; however, when subsequent telephone contact intervention three months post-attempt was compared to a control condition, there was no difference in the number of suicide reattempts between the groups. Other studies on tele-assistance with individuals at risk of suicide have found that monitoring interventions, and specifically telephone follow-up programs, provided promising results and could be considered as a new and useful instrument in the care of those at risk [13,14,15,16,17]. In addition, these interventions incur a relatively low-cost burden to healthcare budgets and can thus be implemented in regions with limited resources.

While there is clearly a need for innovative strategies to treat people who have attempted suicide, patients with schizophrenia have obtained relatively less attention in this regard. The treatment of schizophrenia comprises psychosocial and pharmacological approaches that are generally only mildly effective [18]. Access to care limitations among schizophrenia patients may result in non-adherence to treatment. Telehealth communication services may offer one way of enhancing the stability of treatment response. Digital and mobile applications are in their infancy; currently, they have only been tested in a few trials with marginal quality and inconclusive findings [2].

According to a previous review article [19], modalities including the telephone, Internet and videoconferencing seem to be feasible for schizophrenia patients. Furthermore, preliminary evidence indicates that such approaches appear to enhance patient outcomes [19]. Although telephone follow-up offers a promising tool for increasing treatment adherence after discharge from the hospital, its effectiveness in doing so among this population remains unclear [20].

The present review’s objective was to explore the effectiveness of telephone-delivered, non-pharmacological interventions among patients with schizophrenia and related disorders. To the best of the researchers’ knowledge, no systematic review has been reported that examines telephone-delivered treatments, especially those focused on suicide prevention and the assessment of suicide-specific outcomes, in the schizophrenia patient population.

## 2. Methods

The systematic review was performed in accordance with the Preferred Reporting Items for Systematic Review and Meta-Analysis (PRISMA) [21,22]. We addressed the following research question, constructed according to PICO criteria (Population, Intervention, Comparison and Outcomes) [23]: In adults (≥17 years of age) with schizophrenic spectrum disorders that report prior suicidal behavior (P), what is the effectiveness of telephone-delivered treatments (I) in suicide prevention (O) compared to a control group (C), at any follow-up period?

The inclusion criteria were: (1) adults (≥17 years of age) with a diagnosis of schizophrenia spectrum disorder; (2) presence of suicidal ideation or prior suicidal behavior; and (3) telephone-based suicide prevention interventions (i.e., telephone call, hotline, or text message). The communication should include some, but not necessarily all, of the following elements: improving compliance with medication and follow-up appointments; addressing any problems, stressors, or risk factors; and reducing suicide re-attempts. No restriction was placed on the duration of the intervention.

The exclusion criteria were: (1) presence of non-suicidal self-harm; (2) no comparison condition; (3) non-original studies (e.g., editorials, guidelines, systematic reviews, meta-analyses, protocol); (4) no full-text available.

Literature searches were performed in the PubMed, PsycInfo, Scopus and Web of Science electronic databases. The search strategy yielded articles that contained combinations of the following key terms: (suicide OR self-harm OR self-injury OR self-destructive behavior) AND (schizophrenia OR “psychotic disorder” OR “psychotic symptom” OR “delusional disorder”) AND (telemedicine OR telepsychiatry OR telepsychology OR telephone OR hotline OR helpline). The search terms were selected in accordance with the Peer Review of Electronic Search Strategies guideline statement (PRESS) [24]. The drafted electronic search strategy for each database is included in the Supplementary Materials (Table S1). The search was conducted on 5 September 2022, limited to English or Spanish language, with no restrictions on the publication period. The review methods were established before the review was performed.

The results of the searches were transferred to Mendeley (version 1.19.8). The duplicate articles were automatically removed by Mendeley and manually by the first reviewer (L.C.). The file was blind-screened by two reviewers (A.I.C. and L.C.) using Rayyan Systems Inc. [25] based on titles, abstracts and keywords. The references selected were exported to a template developed by the review group and a blinded screening of the full text was performed to check the eligibility of the study. When necessary, a third reviewer (A.S.) was asked to review the selection process and to resolve discrepancies among the reviewers. The article selection process is described in a PRISMA flow diagram [26].

Two independent authors (A.I.C. and L.C.) extracted data from the selected full-text articles by entering key information from each study into a standardized data extraction template. The general information extracted included the authors, title, research type, date and location of publication, sample size, participant details, intervention and control condition, outcome measures (i.e., instruments and moment of evaluation), feasibility and results.

The risk of bias (RoB) of the selected studies was evaluated employing the Cochrane Collaboration’s Risk of Bias Assessment Tool [27]. We classified studies as high (considered high risk in at least one domain), some concerns (considered raising some concerns in at least one domain), or low risk of bias (considered low risk in all domains).

## 3. Results

The initial search conducted in the four electronic databases revealed a total of 352 candidate publications. Once duplicates were removed, 198 titles, abstracts and keywords were examined, from which 17 studies were retained for full-text review. We excluded any studies that did not focus on the targeted intervention (*n* = 84), did not include a diagnosis of schizophrenia or related disorders (*n* = 30), did not report the presence of suicidal ideation or previous suicidal behavior (*n* = 22), or were not original studies (*n* = 45).

**Figure 1:**
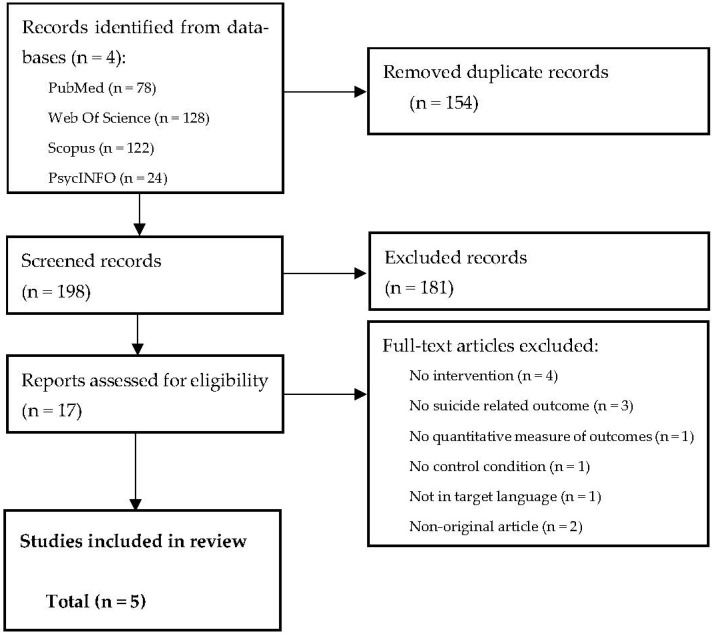
PRISMA flowchart of the study selection process.

A total of 12 studies [3,7,28,29,30,31,32,33,34,35,36,37] were excluded after full-text screening (Supplementary Materials Table S2). We excluded studies that did not refer to telephone-based interventions (*n* = 4) or suicide-related outcomes (*n* = 3). Articles were also ineligible if they did not include a quantitative measurement of suicide-related outcomes (*n* = 1), a comparison condition (*n* = 1), were not written in Spanish or English (*n* = 1), or were not original studies (*n* = 2). Finally, five articles were included in the review (Figure 1). Reviewers 1 and 2 resolved the discrepancies detected and the third reviewer was only required to validate the resulting agreement.

Table 1 summarizes the characteristics of the eligible studies. The sample size oscillated between 7 and 120 in the intervention group (IG) and 6 and 117 in the control group (CG). The average age of the participants was 44.3 (SD = 13.96) and 42.48 (SD = 12.93) in IG and CG, respectively. The percentage of men in all studies was over 90%, both in the IG and CG. A total of three studies assessed veterans diagnosed with schizophrenia or schizoaffective disorder admitted to the healthcare system for suicidal behavior, one focused on patients diagnosed with schizophrenia hospitalized for escalation of suicidality and one considered patients with a primary diagnosis of schizophrenia. The included studies were conducted in two countries: the United States of America (USA; *n* = 4) and China (*n* = 1). All the included study designs are randomized control trials (RCT; *n* = 5). Most of the studies used electronic text messages in addition to face-to-face assessment and telephone calls and only one study involved the participation of a non-specialist healthcare assistant and made use of an electronic platform to deliver text messages. Four studies (80%) implemented the intervention in an inpatient unit [38,39,40,41] and one research work employed a telehealth program in a community setting [42]. The intervention condition was monitored by nursing professionals (*n* = 4; 80%) [38,39,40,41], a non-specialist healthcare assistant (i.e., patient family member or community volunteer) and family physicians or psychiatrists (*n* = 1; 20%) [42]. All studies (*n* = 5) assessed suicidal ideation, 80% (*n* = 4) assessed depressive symptoms, 40% (*n* = 2) assessed positive and negative symptoms and 20% (*n* = 1) assessed treatment adherence. Additionally, four (80%) studies examined the feasibility of the telemonitoring program [39,40,42]. The outcomes were measured with a variety of validated tools (Table 1).

### 3.1. Characteristics of Telehealth Interventions

A total of four articles employed a text-message system as the intervention delivery strategy [38,39,40,41]. Kasckow et al. [38] evaluated a telemonitoring intervention, called Health Buddy© (HB), in a population of US veterans with a diagnosis of schizophrenic disorder recently discharged for suicidal ideation. The HB provided daily monitoring via text-based questions that assisted with symptom evaluations and staff-patient communication. Daily reports were monitored by inpatients nursing staff twice per 8-h shift over a 24-h period. The Beck Scale for Suicidal Ideation (SSI), the Calgary Depression Rating Scale (CDRS), the Clinical Global Impressions and the Scale for Positive Symptoms were administered from baseline to months 1 and 2.

In addition, Kasckow et al. [39] aimed to compare VA Usual Care (UC) with UC augmented with a daily HB program in an RCT. All the participants received a visit within two weeks of being discharged by their clinician and were periodically administered medication. Some participants received more intensive treatment, such as the VA’s Psychosocial Residential Rehabilitation Residential Treatment Program (PRRTP), as part of the intervention for veterans with substance use disorders. Assessment was conducted at baseline and follow-up (weeks 2, 4, 8 and 12) through the SSI and the CDRS.

The same research team, Kasckow et al. [40], investigated whether augmented Intensive Case Monitoring (ICM) with the HB program could produce a significant reduction in suicidal ideation compared to an ICM-only group. The telehealth device connected with participants to ask them questions each day for 10–15 min. The responses were transferred electronically to a secure website and were read by healthcare staff every four hours, seven days a week. The telehealth device targeted the follow-up of symptoms of suicide, depression, psychosis, medication compliance and substance use. The dialogues provided participants with the crisis line phone number in case of escalation in suicidal ideation. To assess the outcomes, the authors used the SSI, the CDRS, the Hamilton Depression Rating Scale (HAMD) and the Positive and Negative Symptoms Scale (PANSS). The study also evaluated the impact of deploying a specialized telehealth case management system employing the HB to reduce suicide among patients with schizophrenia.

More recently, Flaherty et al. [41] hypothesized that adding telehealth to ICM would decrease the number of veterans with schizophrenia or schizoaffective disorder admitted to hospital for psychiatric care in response to suicidal behavior. The telehealth and ICM groups were compared on (1) baseline measures; (2) the number and total days of medical and psychiatric hospitalization 90 days after treatment and (3) the number of ED visits.

Another study performed by Xu et al. [42] examined the impact of adding mobile text messaging to enhance schizophrenia care in a community-based, low-resource setting compared with a community-based free medicine program. The participants in the intervention group received the 686 Program, a national treatment program in which psychiatrists travel twice monthly to the healthcare institution to offer patient consultations and no-cost medication, in addition to LEAN (Lay health supporters, E-platform, Award and iNtegration), a program that included the participation of a non-specialist healthcare assistant and medication reminders, healthcare information and follow-up for relapse with text messaging. The intervention often involved designating a member of the family to supervise the patient’s medication, adverse effects, risk of relapse and requirement for emergency care. While the primary outcomes were treatment adherence, other variables were also measured, including patient symptoms, re-hospitalization and suicide.

Overall, the studies addressing risk management focused on a telehealth monitoring protocol [38,39,40,41]. When participants reported suicidal plans or intent, the nurse contacted a physician, which provided a prompt response. In addition, staff immediately contacted participants when they answered affirmatively to a question about suicidal behavior. If this occurred, healthcare members assessed the situation and determined whether: (1) the participant needed no action; (2) the participant required a consultation with their clinician; or (3) the participant needed to attend the emergency room (ER).

Furthermore, the dialogues assisted patients in determining when to contact their physician in the event of worsening psychotic or depressive symptoms. Patients were also encouraged to adopt recovery-centered behavior, for example, by turning to reliable people in the event of distress. If participants did not upload responses within 24 h, personnel contacted them to confirm their safety and encourage them to continue using the device. The dialogues used were provided by employing patient-centered design methods developed through a panel of consumers and academic experts in suicidality and schizophrenia.

### 3.2. Effectiveness of Telephone-Delivered Interventions for Patients with Diagnosis of Schizophrenia Spectrum Disorders on Suicide Prevention

The telehealth follow-up interventions for suicidal behavior were effective in reducing endpoint suicidal ideation scores compared with baseline in individuals with schizophrenia spectrum disorders relative to a controlled condition [38,39,40].

Kasckow et al. [38] presented results showing a reduction in suicide ideation (*p* = 0.04) along with positive symptoms (*p* = 0.02) in HB patients relative to CG. The preliminary results illustrate that patients reported relatively elevated levels of adherence to HB system; furthermore, telehealth monitoring was associated with earlier remission of suicidal ideation among schizophrenia patients discharged from hospital for suicide treatment.

Consistent with these findings, Kasckow et al. [39] reported significant improvements in SSI scores in HB participants. Overall improvement was shown by both groups in the first 4 weeks. However, mean SSI scores of the HB group patients decreased as time progressed, while for CG patients, these scores decreased initially and then increased after 4 weeks. A significant interaction between time and intervention (F(1, 68) = 5.2; *p* < 0.05) suggests that the HB condition showed a greater enhancement in SSI scores than the CG condition during the 12-week study period. Mean CDRS scores for participants in HB and CG decreased initially and increased over time. No differences were found between HB and CG in depressive symptoms.

Kasckow et al. [40] reported that there were reductions in SSI scores at endpoint compared to baseline in both IG and CG. The SSI scores of the HB group were decreased from 9.8 (SD = 6.15) at baseline to 2.44 (SD = 5.52) at endpoint, while the CG score was decreased from 10.7 (SD = 8.24) at baseline to 2.88 (SD = 6.71) at endpoint. There were no group differences with survival analysis using remission (i.e., SSI score = 0) as the outcome. Nevertheless, for a subgroup with a history of attempted suicide, there was a tendency toward a greater remission at 3 months for those in the HB group (16/18) compared with the CG group (14/19; log rank = 2.82; *df* = 1; *p* = 0.09).

In the study by Flaherty et al. [41], comparisons of treatment conditions disclosed significant differences in hospitalizations. Patients in the telehealth arm had a significantly lower probability than patients in the CG arm of suffering at least one hospitalization (5.0% vs. 32.0%; *p* < 0.05). Moreover, the telehealth condition showed a significantly lower mean number of medical readmissions (0.10 ± 0.45 vs. 0.60 ± 1.19, Mann–Whitney’s U = 4.67; *df* = 1; *p* < 0.05) and number of days of hospitalization (0.70 ± 3.13 vs. 2.56 ± 6.11, Mann–Whitney’s U = 4.59; *df* = 1; *p* < 0.05). No significant group differences were reported in terms of numbers of psychiatric hospitalizations (0.65 ± 1.04 vs. 0.52 ± 0.77) and ED visits (0.60 ± 1.23 vs. 0.92 ± 1.19).

In a community-based study, Xu et al. [42] observed a decline in the risk of relapse (26/120 (21.7%) IG participants vs. 40/117 (34.2%) CG participants; relative risk 0.63 (95% CI 0.42 to 0.97)) and re-hospitalization (9/123 (7.3%) IG participants vs. 25/122 (20.5%) CG participants; relative risk 0.36 (95% CI 0.17 to 0.73)). The program yielded no statistical difference in the other outcomes evaluated, including suicide.

### 3.3. Feasibility

The studies reported that telehealth implementation for the study population appears to be feasible, with adherence and participant evaluation of the intervention as indicators of applicability [39,40]. The application in rural areas, where hospitals did not have specialized services, was also acceptable [42]. Kasckow et al. [38] presented preliminary results revealing relatively favorable adherence to HB use, ranging from 87–100% in the first month to 78–100% in the second month.

Kasckow et al. [39] concluded that telehealth monitoring for patients with schizophrenia discharged from ED for recent suicidal ideation and/or suicide attempt appears to be feasible. The adherence rates for daily use of the HB program were as follows: month_1_ = 86.9% (*n* = 14); month_2_ = 86.3% (*n* = 12); and month_3_ = 84.1% (*n* = 11).

More recently, Kasckow et al. [40] reported monthly adherence for telehealth participants of >80%. Specifically, the monthly adherence rates were as follows: month_1_ = 83% (*n* = 20); month_2_ = 92% (*n* = 19); and month_3_ = 89% (*n* = 15). Post-hoc review of the face-to-face interviews data disclosed that associated factors with mild adherence included: the accentuation of depression (*n* = 2), technical issues (*n* = 4), or distressing home environment (*n* = 2). In addition, 14 of the IG patients answered open-ended questions designed to evaluate the telehealth intervention. For individuals with negative reports, concerns about telehealth monitoring centered on the perceived limitations of the program in treating symptoms, along with its non-personal nature. Concerning positive statements, participants described the intervention as effective in decreasing their suicidal thoughts, improving adherence to medication and reducing anxiety and depressive symptoms.

Along similar lines, Xu et al. [42] reported that the intervention program was generally well-accepted by participants and their family members, while also achieving a good level of participant satisfaction, reporting only 4.3% attrition in comparison to an overall rate of 20% (95% CI 17% to 24%) in other schizophrenia intervention trials [42].

In addition, individual studies pointed out the need to facilitate guidance about how the device was to be used [40,42]. This entailed standard procedures to ensure that participants could use basic phone functions, were able to read/return text messages and understood how information was sent to healthcare professionals.

### 3.4. Strengths and Limitations

Four of the five studies [38,39,40,41] reported that approximately 20% of IG participants never initiated the intervention for the following reasons: technological problems (i.e., lack of mobile device or phone company debt), cognitive difficulties, transportation issues and relapse to substance use.

Focusing on the analysis of the qualitative data, negative reports suggested that patients sometimes experienced frustration when communicating with a machine rather than a human [40]. Despite these negative statements, evaluation of the qualitative data revealed that most of the patients gave positive responses. These favorable statements indicated that the program assisted in instilling hope and that it was useful and easy to use [40].

A further benefit of telehealth monitoring is increased access to care. Enhancing the patient–provider connection via telehealth could improve adherence to treatment. The use of daily monitoring text messages represents another telehealth system approach in the telephone-based intervention spectrum. Individuals with schizophrenia are known to have elevated rates of cognitive impairments and thus a simple and easy-to-use device, as presented in the included studies, could be most appropriate for telemonitoring in this population [39,43,44]. The technology presented allows for ease and efficiency of communication between patients and clinical staff. Therefore, the implementation of a clinical telehealth system shows promise in efforts to address suicide risk among patients at increased risk of recidivism.

Consistent with these findings, Xu et al. [42] reported that the program was found to be reasonably easy to implement and use, incurring only marginal additional costs to patients and their families. The study emphasized the need for adequate training of patients on how to receive, read and respond to text messages, since despite the instructions provided, 28.8% of the participants were still unable to read the messages at the endpoint. To overcome this concern, the researchers delivered voice messages to some participants and, at the same time, sent text messages to the nonprofessional healthcare collaborators. Their experiences warn against the use of smartphones or more complicated applications among individuals with a diagnosis of schizophrenia and a low educational level. Finally, the potential adverse impacts of text messaging on participants and their families deserves further investigation, since 4 (6.3%) patients and 10 (10.7%) non-specialist healthcare assistants indicated that text messaging was bothersome.

### 3.5. Quality of the Studies

Table 2 shows the RoB for each included study. All the studies were randomized trials with a control group, corresponding to the usual mental healthcare provided at the research site (e.g., ICM, 686 Program, or UC plus PRRTP). A methodological concern is that studies relied on convenience samples. Selection bias (i.e., selection of the most compliant participants only) was probably high and compliance rates may have been altered because patients were disposed to consent to the trial, which could have compromised external validity. Therefore, interpretations must be made with care, as the results may not be generalizable to other conditions (i.e., other populations).

## 4. Discussion

The present review is, to our knowledge, the first to summarize studies employing telephone-based interventions for suicide prevention in individuals with schizophrenia. The results of the review suggest that augmenting telehealth is associated with decreased numbers and durations of hospitalizations, appears to be feasible and is associated with the remission of suicidal thoughts among patients with schizophrenia admitted to the hospital for suicide treatment [38,39,40,41]. In rural and resource-poor communities, the delivery of text messages effectively dealt with low compliance rates at marginal cost [30,42]. The interventions were found to be mostly well-accepted by participants and their relatives and were reasonably simple to administer and use. These results could have consequences for the future formulation of clinical policies for the prevention of repeated suicidal behaviors in patients diagnosed with schizophrenia.

A limited number of studies examining the use of telehealth technologies for suicide prevention in patients with schizophrenia or schizoaffective disorder are available. Preliminary research exploring telephone-based modalities have demonstrated that this method appears to be achievable. Furthermore, these preliminary investigations have reported that telehealth monitoring seems to improve outcomes, particularly concerning suicidal ideation, positive symptoms of schizophrenia, treatment adherence, ER visits and medical hospitalization rates [38,39,40,41,42]. Moreover, telephone-based interventions appear to be well-accepted by patients and families [42].

Significant heterogeneity was observed between the included studies, such as year of publication and differences in sample size, in addition to a limited range of languages and nationalities. The article by Kasckow et al. [38] was published in 2009; therefore, it might have a different perspective on telemedicine than the other studies, which were all conducted after 2015. However, the telemedicine program used in the latter studies (Health Buddy©) was comparable.

In addition, the role of the risk of bias assessment in the interpretation of the results should be emphasized. There were several areas of high potential for bias across these studies. The potential for detection bias was high for three studies (60%) [38,39,40], where research staff performing the assessments were not masked. One study (20%) [40] was also judged at high potential for selective reporting bias, largely due to the lack of prior registration of the trial protocol. In summary, the research appears to be at a preliminary stage and improvements in design, sample size and preregistration would be beneficial.

While multiple systematic reviews have been published previously, the participants were not restricted to patients showing suicidal behavior. For example, Kasckow et al. [19] were optimistic about the role of telepsychiatry for the monitoring of patients with schizophrenia. In addition, preliminary evidence suggests that these modalities appear to be feasible and improve patient outcomes, although additional research should focus on further developing interventions and replicating promising results. Gire et al. [45] also conducted a systematic review to evaluate research on mHealth interventions for psychosis. The results hold potential promise for reducing the global gap in mental health treatment by enabling individuals into treatment via their cell phones, particularly those living in remote or rural areas and for those from resource-poor environments. Bright [20] carried out another systematic review of telephone-based interventions in individuals with severe mental illness and concluded that cell phone contact is a potentially beneficial tool for improving medication adherence post-discharge, although their efficacy remains unclear.

While our findings are consistent with previous results, the present report also provides a new perspective that examines telephone interventions implemented in patients with schizophrenia and prior suicidal behaviors. Recently, a pilot trial has been published that evaluated a suicide prevention protocol tailored to patients with psychotic disorders [37]. Shirvastava et al. [7] conducted a community-based study on a crisis helpline targeted at suicide prevention. They reported that the existence of crisis helplines can significantly reduce the delay in treatment and facilitate early and easy access to diagnosis and treatment. Kasckow et al. [35], in a qualitative analysis of the construction of telehealth conversations for the monitoring of suicidal patients with schizophrenia, identified four main issues to be considered when delivering text messages: (1) some themes elicited a high emotional response; (2) there were confidentiality concerns; (3) certain messages were too generalized; and (4) some vocabulary and wording were problematic.

The implementation of a clinical telehealth program offers promise for efforts aimed at addressing suicide risk. The present review adds to the evidence supporting the notion that follow-up is an important element in the management of the disease. Preliminary results indicate that the deployment of the telehealth program is feasible in the follow-up of suicide risk after discharge in patients diagnosed with schizophrenia or schizoaffective disorder. This is relevant for a population that is deemed to be at risk for noncompliance with appointments [46]. Strengthening patient–provider connections through telehealth could increase treatment adherence, help improve access to services for at-risk populations in remote areas and provide opportunities for improving mental health services.

### 4.1. Limitations

Our review has several limitations. A major limitation of the article is the lack of external validity, since the results and conclusions can only be valid in contexts similar to those of the studied research. We identified a limited number of studies conducted to date. In addition, most studies used a small and therefore underpowered sample size, often selecting veterans from a single urban location in the eastern U.S. [38,39,40,41]. The intensive nature of the CG (i.e., two calls and one face-to-face visit from the nurse per week) may have resulted in a marked treatment effect in patients assigned to the control group, which may have obscured any potential improvement from using telehealth as an adjunctive treatment. Moreover, the pursuit of simplicity reduced the capacity to personalize the content, frequency and duration of the messages to patients. While individual adjustment is perceived to be more effective [47], it could also have considerably expanded the complexity and cost of the program. It should be noted that the elevated compliance rates could have been due to selection bias since participants were prepared to consent to the trial, an issue that could have affected external validity [40].

### 4.2. Implication for Research

Evidence for the effectiveness of telephone-based interventions for suicide prevention in patients with schizophrenia spectrum disorders is limited. More RCTs based on clinically representative samples are required, not only to assess compliance, but also to demonstrate maintenance of clinical benefits for participants with schizophrenia, particularly the prevention of suicide reattempts. The selected studies highlighted the need for further research to replicate and amplify the findings, especially considering that the current research used small sample sizes. In addition, the control condition consisted of a considerably more intensive level of intervention than usually offered in outpatient mental health services. The latter is a common problem with studies involving suicidal patients, i.e., achieving the right balance between maintaining sufficient scientific rigor versus keeping high standards of ethics to maximize safety in a high-risk population [40,48]. Determining the adequate CG will be critical in future studies involving telehealth and suicide-risk populations. Further research should also examine the influence of outcomes other than psychopathological measures in facilitating adherence to pharmacological and non-pharmacological treatment.

## 5. Conclusions

Telehealth follow-up for individuals with schizophrenia and suicidal behavior appears to be feasible. The preliminary findings suggest that, in patients with schizophrenia and related disorders, telehealth monitoring drives a larger decrease in suicidal ideation, but not in depressive symptoms, compared to TAU. For the outcome of suicidal deaths, no results were reported to support the presence of a beneficial effect of the intervention since the number of suicide deaths was very low, even in the high-risk ED population. Furthermore, the treatment has not been proven to have a long-term effect. More research is required to expand our knowledge regarding the ways in which telehealth can be used effectively to enhance care for patients with schizophrenic disorders.

The development of telehealth interventions for people with suicidal behavior and schizophrenic disorders is still under construction. The current data provide early indications of the effectiveness of non-pharmacological interventions targeting emotions and cognitive symptoms. Telephone text messaging has proven helpful for strengthening community and family care given its availability, reliability and usability [42]. The telehealth monitoring system further enhanced UC by addressing poor adherence rates at marginal cost. Nevertheless, there is no clear evidence to suggest that texting improves certain clinical symptoms, medication adherence and functioning in patients with schizophrenia [42].

## Figures and Tables

**Table 1:** Participants, interventions, comparisons, outcomes and study designs (PICOS) overview for the included articles (*n* = 5).

Study	Details of Participants	Intervention and Comparison/ Follow-Up Time	Assessment/ Outcome Measure	Results
Kasckow et al., 2009 [38] USA RCT	Patients with schizophrenia recently hospitalized for escalating suicidality; >17 years of age.	Telehealth monitoring group (*n* = 7) vs. ICM (*n* = 6). 24 weeks.	Baseline, 1 and 2 months. Suicidal ideation (SSI), depressive symptoms (CDRS).	Significant improvements in suicidal ideation (*p* = 0.04) in the IG after 2 months.
Kasckow et al., 2015 [39] USA RCT	Veterans with schizophrenia or schizoaffective disorder and recent suicidal ideation and/or suicide attempt; mean age IG = 55.8 (SD = 10.8), mean age CG = 47.5 (SD = 14.7).	Telehealth monitoring group + TAU (*n* = 15) vs. TAU (*n* = 10). 12 weeks.	Baseline, 2, 4, 8 and 12 weeks. Suicidal ideation (SSI), depressive symptoms (CDRS).	Significant improvement in suicidal ideation in the IG during the 12-week study period. No differences between IG and CG in depressive symptoms.
Kasckow et al., 2016 [40] USA RCT	Veterans with schizophrenia or schizoaffective disorder and recent suicidal ideation; mean age IG = 51.0 (SD = 11.7), mean age CG = 51.2 (SD = 11.1).	Telehealth monitoring group + ICM (*n* = 25) vs. ICM (*n* = 26). 13 weeks.	Baseline, 1, 2 and 3 months. Suicidal ideation (SSI), depressive symptoms (CDRS, HAMD).	Reduction in suicidal ideation at endpoint relative to baseline in both groups. For individuals with a history of attempted suicide, there was a tendency for reduced suicidal ideation in IG (*p* = 0.09). No differences were detected for depression.
Flaherty et al., 2017 [41] USA RCT	Veterans with schizophrenia or schizoaffective disorder and suicidal behavior; mean age IG = 49.9 (SD = 12.7), mean age CG = 51.2 (SD = 11.1); IG = 10% female and CG = 4% female.	Telehealth monitoring group + ICM (*n* = 20) vs. ICM (*n* = 25). 13 weeks.	Baseline, 1, 2 and 3 months. Suicidal ideation (SSI), depressive symptoms (CDRS, HAMD), number and days of hospitalizations, number of ER visits.	IG showed a significant reduction compared to CG in medical hospitalization (*p* < 0.05), number of medical admissions (*p* < 0.05) and number of days of medical hospitalization (*p* < 0.05).
Xu et al., 2019 [42] China RCT	Patients with primary diagnosis of schizophrenia; mean age IG = 46.5 (SD = 12.65), mean age CG = 45.5 (SD = 12.72); IG and CG 55.4% female.	LEAN (*n* = 120) vs. TAU (*n* = 117). 26 weeks.	Pre- and post-intervention. Medication adherence (BARS, DAI-10), patient symptoms (CGI), functioning (WHODAS), relapses, re-hospitalization, suicide.	Significant difference between IG and CG for treatment adherence (*p* = 0.013). Significantly lower risk of relapse in IG than CG (21.7% vs. 34.2%) and re-hospitalization (7.3% vs. 20.5%). No statistical difference between IG and CG in suicide outcomes.

BARS: Brief Adherence Rating Scale; CDRS: Calgary Depression Rating Scale; CG: Control Group; CGI: Clinical Global Impression for schizophrenia; DAI-10: Drug Attitude Inventory-10; HAMD: Hamilton Depression Rating Scale; ICM: Intensive Case Monitoring; IG: Intervention Group; LEAN: Lay health supporters, E-platform, Award and iNtegration; PRRTP: Psychosocial Residential Rehabilitation Treatment Program; RCT: Randomized Controlled Trial; SSI: Beck Scale for Suicide Ideation; TAU: Treatment As Usual; USA: United States of America; WHODAS: WHO Disability Assessment Schedule 2.0.

**Table 2:** Risk of bias assessment for each article employing the Cochrane Collaboration’s tool for assessing risk of bias.

Study	Randomization Process	Deviations from Indented Interventions	Missing Outcome Data	Measurement of the Outcome	Selection of the Reported Results	Overall Bias
Kasckow et al., 2009 [38]	Some concerns	Some concerns	Low	High	Some concerns	High
Kasckow et al., 2015 [39]	Low	Some concerns	Some concerns	High	Some concerns	High
Kasckow et al., 2016 [40]	Some concerns	Some concerns	Some concerns	High	High	High
Flaherty et al., 2017 [41]	Low	Some concerns	Some concerns	Some concerns	Some concerns	Some concerns
Xu et al., 2019 [42]	Low	Low	Some concerns	Some concerns	Low	Some concerns

## Data Availability

The data presented in this review are available upon request from the corresponding author.

## Supplementary Materials

The following supporting information can be downloaded at: https://www.mdpi.com/article/10.3390/healthcare11030432/s1, Table S1: Specific search strategy for each database; Table S2: List of articles excluded after full-text screening with the corresponding reason of exclusion.
