# Supplementary material for: Telephone-Delivered Interventions for Suicide Prevention in Schizophrenia and Related Disorders: A Systematic Review
Source: Healthcare (Basel). 2023 Feb 2;11(3):432. doi: 10.3390/healthcare11030432 (PMC9913894; doi:10.3390/healthcare11030432)
Supplement: Supplementary file 1 [file healthcare-11-00432-s001.zip › healthcare-2014391-supplementary.pdf]

healthcare

MDPI

Supplementary Materials

Table S1. Specific search strategy for each database

**PubMed (*n* = 78)**
("suicide"[MeSH Terms] OR suicid*[Title/Abstract] OR "self-injurious behavior"[MeSH Terms] OR self-injur*[Title/Abstract] OR self-harm*[Title/Abstract] OR "self-destructive behavio*"[Title/Abstract] OR self-poisoning[Title/Abstract]) AND ("Schizophrenia"[Mesh Terms] OR schizo*[Title/Abstract] OR "Schizophrenia Spectrum and Other Psychotic Disorders"[Mesh Terms] OR "Psychotic Disorders"[Mesh Terms] OR “psychotic symptom*”[Title/Abstract] OR “delusional disorder*”[Title/Abstract]) AND ("telemedicine"[MeSH Terms] OR telemedicine[Title/Abstract] OR "telecommunications"[MeSH Terms] OR "telecommunication*"[Title/Abstract] OR telehealth[Title/Abstract] OR teleassistance[Title/Abstract] OR telepsychology[Title/Abstract] OR telepsychiatry[Title/Abstract] OR telecare[Title/Abstract] OR telemonitoring[Title/Abstract] OR teleconsult*[Title/Abstract] OR telecounsel*[Title/Abstract] OR "telephone"[MeSH Terms] OR telephon*[Title/Abstract] OR "cell phone"[MeSH Terms] OR phone*[Title/Abstract] OR "phone call"[Title/Abstract] OR "telephone contact*"[Title/Abstract] OR "hotlines"[MeSH Terms] OR hotline*[Title/Abstract] OR "call centers"[MeSH Terms] OR helpline*[Title/Abstract] OR lifeline*[Title/Abstract] OR "crisis line*"[Title/Abstract])
**Web of Science (*n* = 128)**
((TS=(suicid*) OR TS=(“self-injurious behavio$r”) OR TS=(self-injur*) OR TS=(self-harm*) OR TS=(“self-destructive behavio$r”) OR TS=(self-poisoning)) AND (TS=(Schizophrenia) OR TS=(schizo*) OR TS=("Schizophrenia Spectrum and Other Psychotic Disorders") OR TS=("Psychotic Disorders") OR TS=(“psychotic symptom*”) OR TS=(“delusional disorder*”)) AND (TS=(telemedicine) OR TS=(telecommunication*) OR TS=(telehealth) OR TS=(teleassistance) OR TS=(telepsychology) OR TS=(telepsychiatry) OR TS=(telecare) OR TS=(telemonitoring) OR TS=(teleconsult*) OR TS=(telecounsel*) OR TS=(telephon*) OR TS=(“cell phone”) OR TS=(phone*) OR TS=(“phone call*”) OR TS=(“telephone contact*”) OR TS=(hotline*) OR TS=("call center*") OR TS=(helpline*) OR TS=(lifeline*) OR TS=(“crisis line*”)))

**Scopus (*n* = 122)**
(TITLE-ABS-KEY (suicid* OR "self-injurious behavio*" OR self-injur* OR self-harm* OR "self-destructive behavio*" OR self-poisoning) AND TITLE-ABS-KEY (schizophrenia OR schizo* OR "Schizophrenia Spectrum and Other Psychotic Disorders" OR "Psychotic Disorders" OR "psychotic symptom*" OR "delusional disorder*") AND TITLE-ABS-KEY (telemedicine OR telecommunication* OR telehealth OR teleassistance OR telepsychology OR telepsychiatry OR telecare OR telemonitoring OR teleconsult* OR telecounsel* OR telephon* OR "cell phone" OR phone* OR "phone call*" OR "telephone contact*" OR hotline* OR "call center*" OR helpline* OR lifeline* OR "crisis line*"))
**PsycINFO (*n* = 24)**
(MESH(suicide) OR TI(suicid*) OR AB(suicid*) OR IF(suicid*) OR MESH("self-injurious behavior") OR TI(self-injur*) OR AB(self-injur*) OR IF(self-injur*) OR TI(self-harm*) OR AB(self-harm*) OR IF(self-harm*) OR TI("self-destructive behavio*") OR AB("self-destructive behavio*") OR IF("self-destructive behavio*") OR TI(self-poisoning) OR AB(self-poisoning) OR IF(self-poisoning)) AND (MESH("Schizophrenia") OR TI(schizo*) OR AB(schizo*) OR MESH("Schizophrenia Spectrum and Other Psychotic Disorders") OR MESH("Psychotic Disorders") OR TI("psychotic symptom*") OR AB("psychotic symptom*") OR IF("psychotic symptom*") OR TI("delusional disorder*") OR AB("delusional disorder*") OR IF("delusional disorder*")) AND (MESH(telemedicine) OR TI(telemedicine) OR AB(telemedicine) OR IF(telemedicine) OR MESH(telecommunications) OR TI(telecommunication*) OR AB(telecommunication*) OR IF(telecommunication*) OR TI(telehealth) OR AB(telehealth) OR IF(telehealth) OR TI(teleassistance) OR AB(teleassistance) OR IF(teleassistance) OR TI(telepsychology) OR AB(telepsychology) OR IF(telepsychology) OR TI(telepsychiatry) OR AB(telepsychiatry) OR IF(telepsychiatry) OR TI(telecare) OR AB(telecare) OR IF(telecare) OR TI(telemonitoring) OR AB(telemonitoring) OR IF(telemonitoring) OR TI(teleconsult*) OR AB(teleconsult*) OR IF(teleconsult*) OR TI(telecounsel*) OR AB(telecounsel*) OR IF(telecounsel*) OR MESH(telephone) OR MESH(cell phone) OR TI(telephon*) OR AB(telephon*) OR IF(telephon*) OR TI(phone*)

OR AB(phone*) OR IF(phone*) OR TI("phone call*") OR AB("phone call*") OR IF("phone call*") OR TI("telephone contact*") OR AB("telephone contact*") OR IF("telephone contact*") OR MESH(hotlines) OR MESH("call centers") OR TI(hotline*) OR AB(hotline*) OR IF(hotline*) OR TI(helpline*) OR AB(helpline*) OR IF(helpline*) OR TI(lifeline*) OR AB(lifeline*) OR IF(lifeline*) OR TI("crisis line*") OR AB("crisis line*") OR IF("crisis line*"))

Note. No filters activation was executed in databases.

Table S2. List of articles excluded after full-text screening with the corresponding reason of exclusion.

Articles excluded		Reason of exclusion
**Authors**	**Title**	
Palmier-Claus et al., 2013 [28]	Affective Instability Prior to and after Thoughts about Self-Injury in Individuals With and At-Risk of Psychosis: A Mobile Phone Based Study	No telephone-based intervention.
Henson & Torous, 2020 [29]	Feasibility and correlations of smartphone meta-data toward dynamic understanding of depression and suicide risk in schizophrenia	No telephone-based intervention.
Cai et al., 2020 [30]	Mobile Texting and Lay Health Supporters to Improve Schizophrenia Care in a Resource-Poor Community in Rural China (LEAN Trial): Randomized Controlled Trial Extended Implementation	No telephone-based intervention.
Palmier-Claus et al., 2014 [31]	The Temporal Association Between Self-Injurious Thoughts and Psychotic Symptoms: A Mobile Phone Assessment Study	No telephone-based intervention.
Arevian et al., 2020 [32]	Clinical state tracking in serious mental illness through computational analysis of speech	No suicide-related outcomes.
Dausch et al., 2009 [33]	Family-focused therapy via videoconferencing	No suicide-related outcomes.
Smelror et al., 2019 [34]	Feasibility and Acceptability of Using a Mobile Phone App for Characterizing Auditory Verbal Hallucinations in Adolescents With Early-Onset Psychosis: Exploratory Study	No suicide-related outcomes.
Kasckow et al., 2014 [35]	Development of Telehealth Dialogues for Monitoring Suicidal Patients with Schizophrenia: Consumer Feedback	No quantitative measure of outcomes.
Shrivastava et al., 2012 [7]	Reducing treatment delay for early intervention: evaluation of a community based crisis helpline	No comparison condition.
Böcker, 1984 [36]	Soziale Integration und Kontakte zu Bezugspersonen des gewohnten sozialen Umfeldes wahrend stationairer Behandlung im psychiatrischen Krankenhaus	Not written in Spanish or English.
Depp et al., 2021 [37]	A Brief Mobile-Augmented Suicide Prevention Intervention for People With Psychotic Disorders in Transition From Acute to Ongoing Care: Protocol for a Pilot Trial	Non-original studies
Kasckow et al., 2011 [3]	A telehealth intervention for suicidal patients with schizophrenia	Non-original studies
